# Supplementary material for: Gut microbiota structure differs between honeybees in winter and summer
Source: ISME J. 2019 Dec 13;14(3):801–14. doi: 10.1038/s41396-019-0568-8 (PMC7031341; doi:10.1038/s41396-019-0568-8)
Supplement: Supplementary file 1 — Summary of Supplementary Information [file 41396_2019_568_MOESM1_ESM.pdf]

Summary of Supplementary Information:

Supplementary Figures are included in Separate files as Fig S1, Fig S2, Fig S3, Fig S4, Fig S5.
Supplementary figure legends can be found in Supplementary_Figure_Legends.PDF
Supplementary Tables S1-S7 can be found in the folder Supp.Tables.xlsx
